# Combination Strategies to Augment Immune Check Point Inhibitors Efficacy - Implications for Translational Research

**DOI:** 10.3389/fonc.2021.559161

**Published:** 2021-05-28

**Authors:** Hrishi Varayathu, Vinu Sarathy, Beulah Elsa Thomas, Suhail Sayeed Mufti, Radheshyam Naik

**Affiliations:** ^1^ Department of Translational Medicine and Therapeutics, HealthCare Global Enterprises Limited, Bangalore, India; ^2^ Department of Medical Oncology, HealthCare Global Enterprises Limited, Bangalore, India; ^3^ Department of Clinical Pharmacology, HealthCare Global Enterprises Limited, Bangalore, India

**Keywords:** translational research, immune checkpoint inhibitors (ICI), preclinical model, clinical trials, combination strategies, immunotherapy adjuncts

## Abstract

Immune checkpoint inhibitor therapy has revolutionized the field of cancer immunotherapy. Even though it has shown a durable response in some solid tumors, several patients do not respond to these agents, irrespective of predictive biomarker (PD-L1, MSI, TMB) status. Multiple preclinical, as well as early-phase clinical studies are ongoing for combining immune checkpoint inhibitors with anti-cancer and/or non-anti-cancer drugs for beneficial therapeutic interactions. In this review, we discuss the mechanistic basis behind the combination of immune checkpoint inhibitors with other drugs currently being studied in early phase clinical studies including conventional chemotherapy drugs, metronomic chemotherapy, thalidomide and its derivatives, epigenetic therapy, targeted therapy, inhibitors of DNA damage repair, other small molecule inhibitors, anti-tumor antibodies hormonal therapy, multiple checkpoint Inhibitors, microbiome therapeutics, oncolytic viruses, radiotherapy, drugs targeting myeloid-derived suppressor cells, drugs targeting Tregs, drugs targeting renin-angiotensin system, drugs targeting the autonomic nervous system, metformin, etc. We also highlight how translational research strategies can help better understand the true therapeutic potential of such combinations.

## Introduction

Immunotherapy is often thought to be a recent discovery. However, if we look at the beginnings of cancer immunotherapy, the first scientific attempts were made Fehleisen and Busch, German physicians who noted significant tumor regression post erysipelas infection. William Bradley Coley, the father of immunotherapy first tried to use the immune system in 1981 for the treatment of bone cancer. His work was largely ignored for more than fifty years when the field of immunology took off with the discovery of T cell existence and their role in immunity. In the 1970s, Donald L. Morton treated melanoma patients with Bacillus Calmette-Guerin (BCG) and 91% of patients treated had disease regression. Then in the 1980s, the first immunotherapy cancer treatment IL-2 was approved for the treatment of melanoma and kidney cancer by FDA (1). The most significant breakthrough in immunotherapy came through James Allison & Tasuku Honjo who discovered CTLA4 and PDL1 respectively as therapeutic targets. That research work has further led to the successful development of new checkpoint inhibitors, Dendritic cell therapy, CAR-T cells, and other adoptive cell therapies, oncolytic viruses which have all brought hope to the future of immunotherapy (2).

PD1 and CTLA-4 are the two distinct inhibitory receptors on T cells, which are targeted by monoclonal antibodies for providing a durable antitumor immune response. The efficacy of immune checkpoint inhibitors (ICI) was first noted in metastatic melanoma with anti-tumor immune response and significant improvement in overall survival (OS) of patients received ipilimumab an anti-CTLA4 antibody (3). This led to the accelerated clinical studies using anti-PD1/PDL-1 antibodies and regulatory approval in other malignancies including few hematological malignancies, non-small cell lung cancer (NSCLC), and melanoma (4, 5). Currently, six ICIs have been approved by the US FDA, of which five ICIs also received market authorization by the European Medicines Agency (EMA).

Even though these therapies have shown survival benefits with reduced incidence of drug resistance or adverse effects than conventional chemotherapy, not all cancer patients are benefitted from these drugs (6). The ability of the tumor microenvironment (TME) to evade immune responses and thereby generating immunosuppression/immune escape is one of the prime challenges that remain (7). This tumor-derived local environment is known as the immunosuppressive TME which ultimately suppresses the antitumor immune response. Clinical trial data analysis of ICI reveals mainly three kinds of patient response: (a) patients who do not respond initially (innate resistance); (b) those who respond at first but fail subsequently (acquired resistance); and (c) those continuously respond from initially to later stages (8, 9). Antigen experienced T-cell activation and proliferation are entailed for the generation of an effective antitumor response. The difference in response is because of the insufficient generation and activity of anti-tumor CD8 T-cells (10). Impaired processing and presentation of neo antigens are other causes for the lack of activation of tumor-reactive T cells. Multiple other factors like type of cancer, tumor heterogeneity, multiple lines of treatment, and the immunosuppressive TME generated due to tumor-related and non- tumor related factors result in poor response to ICIs (11). Another important obstacle in reduced ICI efficacy is tumor associated hypoxia due to the structural organization of stroma. This can results in altering the pharmacokinetics of the drug by reducing the target drug disposition. Hypoxia can potentiate tumor invasion, stemness, and metastatic capacity mainly through the activation of hypoxia-inducible factor-1α– mediated hepatocyte growth factor/c-Met pathway (12, 13). Immunosuppressive microenvironment-associated macrophages enhance tumor hypoxia and further reduce the efficacy of ICIs (14). These results highlight the necessity of combination therapies that can improve the tumor immunogenicity, changing the immunosuppressive TME and targeting other pathways which potentially inhibit the activation of T cells. There are many drugs including cytotoxic as well as non-anti-cancer drugs which have shown promising results in potentiating the efficacy of ICIs.

## Materials and Methods

**Figure 1:**
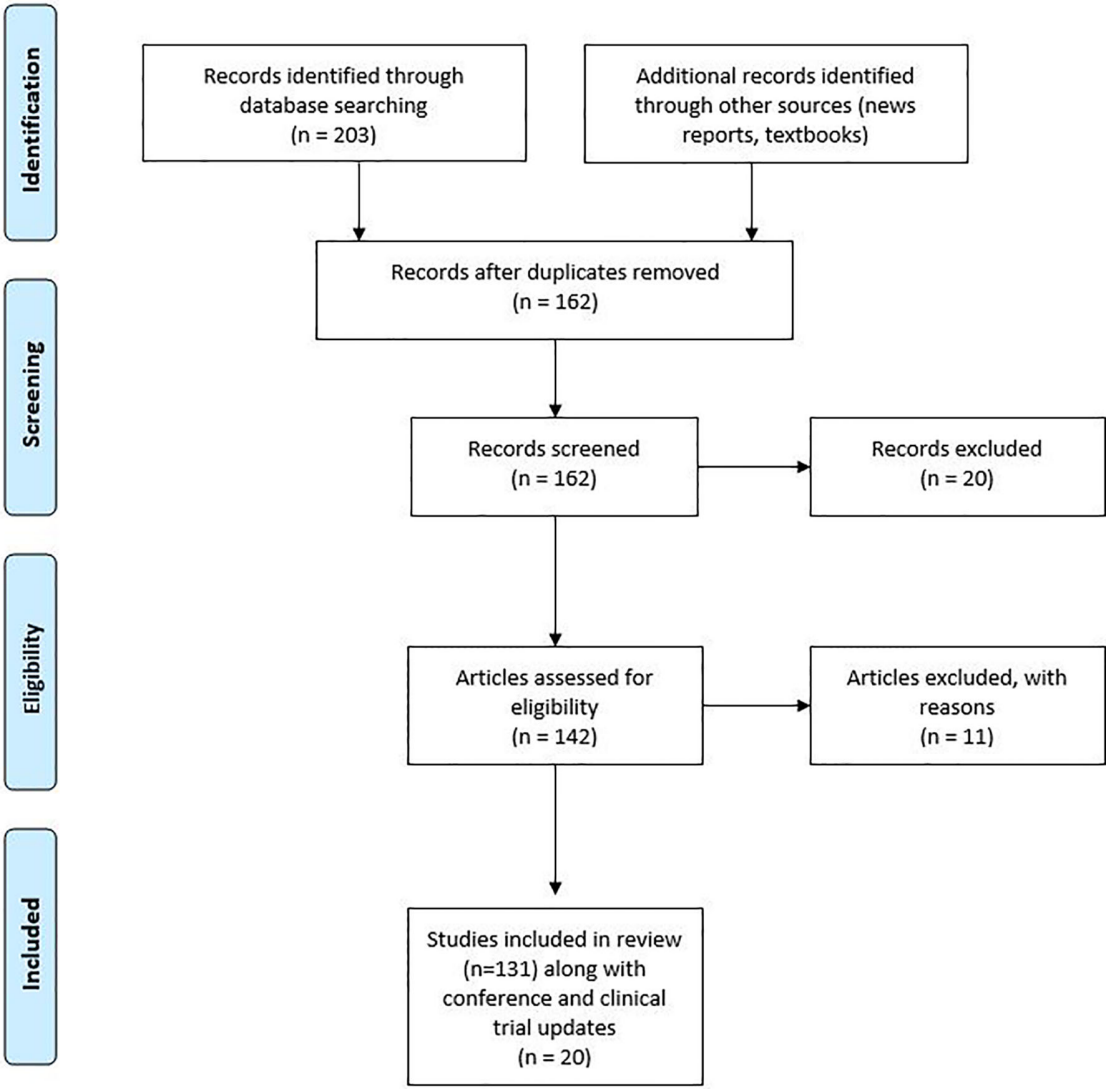
Research strategy with PRISMA flow diagram.

We collected 250 published studies that had used the combination of ICIs and other therapies. From the collected studies duplicates were removed and further assessed for eligibility as shown in **Figure 1**. The review covered all countries with a web-based search of PubMed/Google Scholar published from 2005 to 2019. A further search was conducted in ClinicalTrials.gov to check for the available clinical trials. A few of the clinical trials were further explored by contacting the study teams or information gathered from recent press releases for the study.

### Inclusion/Exclusion Criteria

Original primary research such as experimental, observational, and qualitative studies which are written in English and published in peer-reviewed journals are included in the review. Some of the review articles were also selected if they contained outcomes of previous exploratory studies. Search terms were used to find specific studies related to the subject (**Table 1**). Articles that define the mechanism behinds the use of drugs as adjuncts with ICIs were considered eligible.

**Table 1:** Search terms used for the collection of articles.

Immune therapy	Enhancer
‘Immune checkpoint Inhibitors’, ‘anti-PD(L)1’, ‘anti CTLA4’	“Chemotherapy” OR “metronomic chemotherapy”
‘Immune checkpoint Inhibitors’, ‘anti-PD(L)1’, ‘anti CTLA4’	“Epigenetic therapy” OR “hypomethylating agents” OR “Histone deacetylase inhibitors”
‘Immune checkpoint Inhibitors’, ‘anti-PD(L)1’, ‘anti CTLA4’	“Anti-angiogenic therapies” OR “Tyrosine Kinase Inhibitors” OR “Bevacizumab”
‘Immune checkpoint Inhibitors’, ‘anti-PD(L)1’, ‘anti CTLA4’	“Microbiota” OR “gut microbe” OR “microbiome”
‘Immune checkpoint Inhibitors’, ‘anti-PD(L)1’, ‘anti CTLA4’	“Oncolytic Virus” OR “Talimogene Laherparepvec” OR “Coxsackie virus”
‘Immune checkpoint Inhibitors’, ‘anti-PD(L)1’, ‘anti CTLA4’	“Targeted therapy” OR “CDK4/6 Inhibitors” OR “PARP Inhibitors” OR “Other small molecule inhibitors” OR “Bruton Kinase Inhibitors” OR “selective estrogen down regulators”
‘Immune checkpoint Inhibitors’, ‘anti-PD(L)1’, ‘anti CTLA4’	“Radiation” OR “Radiotherapy” OR “abscopal effect”
‘Immune checkpoint Inhibitors’, ‘anti-PD(L)1’, ‘anti CTLA4’	“T cell co-stimulator” OR “Nectin 4” OR “Enfortumab Vedotin” OR “Interleukin 6”
‘Immune checkpoint Inhibitors’, ‘anti-PD(L)1’, ‘anti CTLA4’	“Drugs targeting renin-angiotensin system OR Losartan”
‘Immune checkpoint Inhibitors’, ‘anti-PD(L)1’, ‘anti CTLA4’	“Drugs targeting autonomous nervous system” OR “Beta Blockers”
‘Immune checkpoint Inhibitors’, ‘anti-PD(L)1’, ‘anti CTLA4’	“Oral hypoglycemic agents” OR “Metformin” OR “Rosiglitazone” OR “pioglitazone”
‘Immune checkpoint Inhibitors’, ‘anti-PD(L)1’, ‘anti CTLA4’	“Drugs targeting Myeloid-derived suppressor cells” and Tregs”

We mainly focused on the mechanisms and rationale of using an adjunct with ICIs for the combinations already in clinical trials. Adjuncts used only in preclinical studies with no currently available clinical data have been excluded from the review.

### Anti-Cancer Drugs in Combination With ICIs

#### Cytotoxic Drugs

Conventional chemotherapy drugs such as anthracyclines, cyclophosphamide, Cisplatin, Oxaliplatin, gemcitabine, temozolomide, and paclitaxel are observed to enhance tumor immunity at lesser doses. These drugs at normal dosage and schedule produce immunogenic cell death (ICD) which results in the tumor antigen release and danger signal generation from dying cancer cells known as damage-associated molecular patterns (DAMPs), which in turn provide tumor-targeting immune responses (15). The immunogenicity of tumor cells is modulated by these drugs *via* various mechanisms comprising (1) enhanced tumor antigen expression and presentation; (2) down regulating coinhibitory molecules (B7-H1/PDL1) and upregulating costimulatory molecules (B7-1) expressed on the surface of tumor cells which in turn increases the function of effector T-cell; (3) granzyme and perforin-dependent mechanisms increasing T-cell facilitated tumor cell lysis; and (4) decreasing MDSCs and Tregs infiltration in the TME.

In mouse immunization experiments using tumor cells pretreated with chemotherapeutic agents such as mitoxantrone and doxorubicin, effective cancer regression *via* CRT (calreticulin) expression and HMGB1 secretion was observed (16, 17). These findings have been confirmed in clinical studies which show that CRT expression is crucial for better prognosis in cancer patients (18). Many studies over the past decade have shown that different chemotherapy regimens also promote differentiation of Th1/Th2 and amplify the proliferation of T-lymphocytes in solid cancers (renal cell carcinoma, colon cancer, and ovarian cancer) (19, 20).

Various clinical trials such as KEYNOTE-189, IMPower-132 which used ICI combination with chemotherapy in non-squamous NSCLC have shown significant benefits.

After a median follow-up of 18.7 months, the combination of pembrolizumab and chemotherapy resulted in a longer OS compared to chemotherapy alone, with a median OS of 22.0 months versus 10.7 months (hazard ratio [HR], 0.56; 95% CI, 0.45-0.70, P.00001). At 9.0 months versus 4.9 months, respectively, progression-free survival (PFS) was also substantially increased as compared to placebo plus chemotherapy (HR, 0.48; 95% CI, 0.40-0.58, P <.00001).

Recent findings from KEYNOTE-062 which used first-line ICI chemotherapy combination in advanced gastric or gastroesophageal junction (G/GEJ) adenocarcinoma was non-inferior to chemotherapy and showed a slight improvement in OS. Pembrolizumab plus chemotherapy resulted in a median OS of 12.5 months (95% CI, 10.8-13.9) versus 11.1 months (95% CI, 9.2-12.8) with chemotherapy (HR 0.85; 95% CI, 0.70-1.03; p = 0.046) (21–23).

The second interim analysis of IM passion 130 trial that assessed the efficacy and safety of atezolizumab plus nab-paclitaxel in patients with unresectable, locally advanced, or metastatic triple-negative breast cancer (TNBC) reported that median OS analysis in patients with PD-L1 immune cell-positive tumors was 25 months (95% CI 19.6–30.7) in atezolizumab + nab-paclitaxel arm and 18 months (13.6–20.1) in placebo + nab-paclitaxel arm(stratified HR 0.71, 0.54–0.94]) (24).

In March 2020, FDA approved Durvalumab chemotherapy (Etoposide + Cisplatin or carboplatin) combination as a first-line treatment for extensive-stage small cell lung cancer (ES-SLC). In untreated previously ES-SLC, CASPIAN trial showed a median OS of 13 months (95% CI: 11.5, 14.8) in the durvalumab plus chemotherapy group compared with 10.3 months (95% CI: 9.3, 11.2) in the chemotherapy alone arm (hazard ratio 0.73; 95% CI: 0.59, 0.91; p=0.0047). However, median PFS was almost the same in the combination arm (5.1months) vs chemotherapy alone (5.4months) arm (25).

##### Metronomic Therapy

Metronomic therapy, an alternate approach for chemotherapy administration encompasses continuous administration of lesser doses of cytotoxic agents. Various studies have reported significant anti-tumor immune responses, lower therapeutic resistance, and a decrease in tumor vascularization (26–28). Chemotherapeutic drugs such as methotrexate, cyclophosphamide, etoposide, vinblastine, and paclitaxel are used widely as metronomic chemotherapy in various cancers. Even though high dose chemotherapy target cancer cells and are immunosuppressive, high-frequency low dose chemotherapy is considered to be immunostimulatory by targeting the supporting tumor stroma. Cancer cells in tumors cause the surrounding stroma to release additional angiogenesis stimulators. Angiogenesis in the tumor is a process initiated by growth factors and tumor-associated endothelial cells (TEC). When exposed to cancer chemotherapeutic agents at much lower concentrations than those needed to cause tumor cell damage, TECs lose functionality. This property of low-dose chemotherapy such as vinblastine, doxorubicin, paclitaxel, and carboplatin is documented in many studies (29, 30). This can reduce the tumor-resistant clones, by giving combination with other treatment modalities (31).

Chemotherapy administered metronomically reduces the number of immunosuppressive regulatory T cells (Tregs) (32–34), promote antigen-presenting cell maturations (35), improves the activity of dendritic cells (DCs) (36) and, chiefly enhances the activation and function of cytotoxic CD8+ T cells and NK cells. Moreover, it also targets other TME immunosuppressive components referred to as myeloid-derived suppressor cells (MDSCs) (37–40). MDSCs are a diverse group of myeloid immune cells characterized by their immature state and ability to suppress T cells. This particular property of metronomic chemotherapy can be used for the modulation of the efficacy of ICIs and many studies have reported positive results. It is proposed that metronomic chemotherapy promotes tumor-specific immune activation; concurrent administration of ICIs could maintain the T cells in an activated state. Combination of low dose cyclophosphamide and oxaliplatin sensitized tumor ICI which are initially refractory by increasing CTLs/Treg ratio in the TME in a murine model of lung adenocarcinoma (41). Similar results were obtained in the murine colorectal cancer model where oxaliplatin augmented the amounts of CTLs and activated dendritic cells (DCs) potentiating the efficacy of anti-PD-L1 therapy (42). Enhanced infiltration of CD8+ and CD4+FoxP3T along with suppression of the CD4+ CD25+ FoxP3+ regulatory T cell function have seen after low dose cyclophosphamide with an anti-PD1 agent and improved tumor-free survival in a model of cervical cancer (43, 44). There are clinical studies ongoing, combining a metronomic dose of cyclophosphamide along with ICIs as depicted in **Table 2**.

**Table 2:** Few clinical trials listed in Clinical trials.gov which uses combination strategies with ICIs as of 17-04-2020.

Adjunct Therapy	ICI used	Study Title	Clinical Trial Reference	Phase	Status
Metronomic Vinorelbine	Atezolizumab	Trial to Evaluate Safety and Efficacy of Vinorelbine With Metronomic Administration in Combination With Atezolizumab as Second-line Treatment for Patients With Stage IV NSCLC (VinMetAtezo)	NCT03801304	Phase 2	Recruiting
Metronomic Cyclophosphamide	Pembrolizumab	Evaluation of Pembrolizumab in Lymphopenic Metastatic Breast Cancer Patients Treated With Metronomic Cyclophosphamide (CHEMOIMMUNE)	NCT03139851	Phase 2	Active
Methotrexate, Etoposide, Ifosfamide, Dexamethasone, Pegaspargase	Pembrolizumab	Chemoimmunotherapy and Allogeneic Stem Cell Transplant for NK T-cell Leukemia/Lymphoma	NCT03719105	Phase 1	Recruiting
Nab-Paclitaxel, Epirubicin, Cyclophosphamide	Pembrolizumab	Neoadjuvant Study of Pembrolizumab in Combination With Nab-paclitaxel Followed by Pembrolizumab in Combination With Epirubicin and Cyclophosphamide in Patients With Triple-Negative Breast Cancer	NCT03289819	Phase 2	Active
Carboplatin, Etoposide	Atezolizumab	A Study of Atezolizumab in Combination With Carboplatin Plus Etoposide to Investigate Safety and Efficacy in Patients With Untreated Extensive-Stage Small Cell Lung Cancer (MAURIS)	NCT04028050	Phase 3	Recruiting
Pegylated liposomal doxorubicin, Cyclophosphamide	Atezolizumab	Atezolizumab Combined With Immunogenic Chemotherapy in Patients With Metastatic Triple-negative Breast Cancer (ALICE)	NCT03164993	Phase 2	Recruiting
5-Fluorouracil, Irinotecan, Leucovorin calcium, Oxaliplatin	Nivolumab	Nivolumab in Combination With Chemotherapy Before Surgery in Treating Patients With Borderline Resectable Pancreatic Cancer	NCT03970252	Phase 2	Recruiting
Capecitabine, Carboplatin, Gemcitabine Hydrochloride, Nab-paclitaxel, Pegylated Liposomal Doxorubicin Hydrochloride	Durvalumab	Durvalumab in Combination With Chemotherapy in Treating Patients With Advanced Solid Tumors, (DURVA+ Study)	NCT03907475	Phase 2	Recruiting
ATRA	Pembrolizumab	Pembrolizumab and All-Trans Retinoic Acid Combination Treatment of Advanced Melanoma	NCT03200847	Phase 2	Recruiting
Lenalidomide	Nivolumab	Nivolumab and Lenalidomide in Treating Patients With Relapsed or Refractory Non-Hodgkin or Hodgkin Lymphoma	NCT03015896	Phase 2	Recruiting
Lenalidomide, Pomalidomide, Daratumumab, Dexamethasone	Atezolizumab	A Study of Atezolizumab (Anti-Programmed Death-Ligand 1 [PD-L1] Antibody) Alone or in Combination With an Immunomodulatory Drug and/or Daratumumab in Participants With Multiple Myeloma (MM)	NCT02431208	Phase 1	Active
Vorinostat, Tamoxifen	Pembrolizumab	Reversing Therapy Resistance With Epigenetic-Immune Modification	NCT02395627	Phase 2	Active
Vorinostat	Pembrolizumab	Pembro and Vorinostat for Patients With Stage IV NSCLC	NCT02638090	Phase 2	Recruiting
Guadecitabine	Atezolizumab	A Study Evaluating the Safety and Pharmacology of Atezolizumab Administered in Combination With Immunomodulatory Agents in Participants With Acute Myeloid Leukemia (AML)	NCT02892318	Phase 1	Completed
Azacitidine	Pembrolizumab	Azacitidine and Pembrolizumab in Pancreatic Cancer	NCT03264404	Phase 2	Recruiting
Pegylated liposomal doxorubicin, Cyclophosphamide	Nivolumab, Ipilimumab	Phase IIb Study Evaluating Immunogenic Chemotherapy Combined With Ipilimumab and Nivolumab in Breast Cancer (ICON)	NCT03409198	Phase 2	Recruiting
Selicrelumab, Cobimetinib, Gemcitabine + Carboplatin or Eribulin, Capecitabine, Bevacizumab, Ipatasertib	Atezolizumab	A Study Evaluating the Efficacy and Safety of Multiple Immunotherapy-Based Treatment Combinations in Patients With Metastatic or Inoperable Locally Advanced Triple-Negative Breast Cancer (Morpheus-TNBC) (Morpheus-TNBC)	NCT03424005	Phase 2	Recruiting
Trabectedin	Nivolumab, Ipilimumab	SAINT: Trabectedin, Ipilimumab, and Nivolumab as First-Line Treatment for Advanced Soft Tissue Sarcoma	NCT03138161	Phase 2	Recruiting
Trabectedin	Nivolumab	Combined Treatment With Nivolumab and Trabectedin in Patients With Metastatic or Inoperable Soft Tissue Sarcomas (NiTraSarc)	NCT03590210	Phase 2	Recruiting
Talimogene Laherparepvec (Oncolytic Virus), Trabectedin	Nivolumab	Talimogene Laherparepvec, Nivolumab, and Trabectedin for Sarcoma (TNT)	NCT03886311	Phase 2	Recruiting
Letrozole, Palbociclib	Pembrolizumab	Pembrolizumab, Letrozole, and Palbociclib in Treating Postmenopausal Patients With Newly Diagnosed Metastatic Stage IV Estrogen Receptor-Positive Breast Cancer	NCT02778685	Phase 2	Recruiting
Entinostat, Fulvestrant, Ipatasertib, Exemestane, Tamoxifen, Abemaciclib	Atezolizumab	A Study of Multiple Immunotherapy-Based Treatment Combinations in Hormone Receptor (HR)-Positive Human Epidermal Growth Factor Receptor 2 (HER2)-Negative Breast Cancer (MORPHEUS HR+BC)	NCT03280563	Phase 2	Recruiting
Cabozantinib	Ipilimumab, Nivolumab	Combined Immunotherapy and Targeted Therapy for Hepatocellular Carcinoma	NCT01658878	Phase 2	Active
Pemetrexed, Carboplatin, Bevacizumab	Atezolizumab	Carboplatin Plus Pemetrexed Plus Atezolizumab Plus Bevacizumab in Chemotherapy and Immunotherapy-naïve Patients With Stage IV Non-squamous NSCLC	NCT03713944	Phase 2	Recruiting
Radiation Therapy	Pembrolizumab	Pembrolizumab With or Without Radiation in Patients With Recurrent or Metastatic Adenoid Cystic Carcinoma	NCT03087019	Phase 2	Active
Decitabine, Radiation Therapy	Pembrolizumab	Pembrolizumab in Combination With Decitabine and Hypofractionated Index Lesion Radiation in Pediatrics and Young Adults	NCT03445858	Phase 2	Recruiting
Dasatinib or Imatinib Mesylate or Nilotinib	Pembrolizumab	Pembrolizumab and Dasatinib, Imatinib Mesylate, or Nilotinib in Treating Patients With Chronic Myeloid Leukemia and Persistently Detectable Minimal Residual Disease	NCT03516279	Phase 2	Recruiting
Multiple ICIs	Durvalumab, Tremelimumab	Neoadjuvant Immunotherapy With Durvalumab and Tremelimumab for Bladder Cancer Patients Ineligible for Cisplatin (NITIMIB)	NCT03234153	Phase 2	Active
Multiple ICIs	Pembrolizumab, Ipilimumab	Low Dose Ipilimumab With Pembrolizumab in Treating Patients With Melanoma That Has Spread to the Brain	NCT03873818	Phase 2	Active
Ibrutinib	Pembrolizumab	Pembrolizumab in Combination With Ibrutinib for Advanced, Refractory Colorectal Cancers	NCT03332498	Phase 2	Active
Ibrutinib, Cetuximab	Nivolumab	Trial of Ibrutinib Combined With Nivolumab or Cetuximab to Treat Recurrent/Metastatic HNSCC	NCT03646461	Phase 2	Recruiting
Fecal Microbiota transplant	Pembrolizumab	Fecal Microbiota Transplant and Pembrolizumab for Men With Metastatic Castration-Resistant Prostate Cancer.	NCT04116775	Phase 2	Recruiting

##### Thalidomide and Its Derivatives

Thalidomide was first used for its direct anti-tumor actions on myeloma cells by resulting in cell cycle arrest and anti-angiogenic effect. Thalidomide derivatives such as lenalidomide, pomalidomide were then classified as immunomodulatory agents as they stimulate T cells and natural killer cells (NK) to secrete IL-2 and IFNγ and inhibit Tregs. This resulted in the amplification of specific immunity in myeloma (45, 46). Lenalidomide in combination with dexamethasone and proteasome inhibitor (Bortezomib and Carfilzomib) is considered as a standard chemotherapy regimen for multiple myeloma. Some studies show that the immunostimulatory effects of lenalidomide are hampered by the concurrent use of dexamethasone (46). The immunomodulatory effects of these drugs can be exploited to enable an enhanced action with ICIs. An invitro cell line study suggests good results with the combination of ICIs and thalidomide derivatives in anti-leukemic therapy (47).

Despite these effects, FDA on July 2017 placed a clinical hold on three clinical trials of the programmed death 1 (PD-1) inhibitor pembrolizumab in combination with lenalidomide or pomalidomide for patients with multiple myeloma: KEYNOTE-183, KEYNOTE-185, and KEYNOTE-023 because of increasing reports of death. However, there are similar clinical trials ongoing with the combination of ICIs and lenalidomide for other indications such as lymphoma (47–49). In KEYNOTE-023 responses were durable in the overall study population which includes lenalidomide refractory patients with a median duration of response (DOR) of 18.7 months, Objective response rate (ORR) of 44%. Median PFS was 7.2 months and median OS was not reached (50). The median PFS was 27.6 months with a median follow-up of 32.2 months in a two-year update of a Phase II trial of Pembrolizumab, Lenalidomide, and dexamethasone as post autologous stem cell transplant consolidation in multiple myeloma. Similarly, Badros et al. reported ORR-60%, DOR 14.7 months, median PFS 17.4 months, Median OS not reached with Pembrolizumab, pomalidomide and dexamethasone combination and they also reported long term remissions after discontinuing Pembrolizumab, 57% have ongoing response without any further treatment with a median survival of 18 months (51, 52).

#### Epigenetic Therapy

##### Hypomethylating Agents

Hypomethylating agents such as azacytidine and decitabine are mainly used in the treatment of myelodysplastic syndromes (MDS) and acute myeloid leukemia (AML). Due to the availability of advanced genomic studies, it is evident that hypermethylation of tumor suppressor genes in the promoter region is one of the pathways of carcinogenesis (53). The most common and stable of epigenetic alterations in cancer is methylation of DNA at CpG islands. Hypermethylation tends to limit ICIs by blocking endogenous interferon responses required for cancer cell recognition whereas global hypomethylation marks in the expression of inhibitory cytokines and PD-L1, supplemented by epithelial-mesenchymal variations contribute to immunosuppression. The drivers of these opposing states of methylation are not stated well. DNA methylation is also essential in the ‘exhaustion’ of cytotoxic T cells that occurs as a result of tumor progression (54). The feasibility of targeting this mechanism with hypomethylating agents is being widely studied now. Other than this, the collective data of studies suggests that hypomethylating agents are known to promote the expression of cancer-specific antigens and major histocompatibility complex (MHC) results in improved immunologic recognition of cancer cells and enhanced anti-tumor responses (55–57). This property is exploited By Brahmer J et al. and when six patients in a clinical trial of epigenetic therapy for advanced treatment-resistant NSCLC were switched to an immunotherapy trial targeting the PD-1/PD-L1 immune tolerance checkpoint, they saw a substantial improvement. Three of the six patients had long-term partial responses to immunotherapy, ranging from 14 to 26 months, while the other two had stable disease for 8.25 and 8.5 months, respectively (4, 5, 58). Various clinical studies are ongoing to check the efficacy and toxicity of this combination therapy in various cancers (**Table 2**).

##### Histone Deacetylase Inhibitors (HDACis)

As discussed above, in cancers that are immune to immunotherapy, epigenetic inhibitors may be used as adjunctive therapy or as a replacement for immunotherapy (sole agents of immune modification). In the TME, epigenetic changes are normal, resulting in a variety of gene expression changes and tumor escape. Recently, HDAC inhibitors have shown potential anti-cancer properties such as cell cycle arrest, anti-angiogenesis, activation of both intrinsic and extrinsic apoptotic pathways, autophagy, and modulation of immune responses. HDACs are classified mainly into 4 categories (Class I, Class II, Class III, Class IV) based on their structure, which is further subdivided into Zinc dependent (Class I, Class II, Class IV) and NAD+ dependent (Class III) for their enzymatic activity (59). Modulation of the immune response can be utilized for improving the outcome of ICIs. HDACis can change the expression of immune system upregulaters including MHC and costimulatory molecules, which affects antigen presentation and thus T cell activation. It has also been observed that naïve T cell functionality is stimulated by HDAC6i and Tregs are targeted by class II HDACis. The Class I HDACis target adaptive immunity by enhancing natural killer and CD8 cells functionality. Several non-selective HDACis such as vorinostat, belinostat, trichostatin A are being tried in clinical trials (60). HDAC I, II, and IV are inhibited by Pan-HDACi such as valproic acid, vorinostat, and panobinostat. MS275 (a class I HDAC inhibitor), MC1568 (a class III HDACi), sirtinol (a class II HDACi), and Nexturastat A (HDAC6i), among many others, are currently being used to reduce the toxic effects associated with pan-HDAC inhibition. The wide range of biological effects may be due to each inhibitor’s particular chemical structure and mechanistic profiles (61).

In a B16F10 syngeneic murine model study by Wood DM et al. HDAC inhibitor treatment resulted in rapid upregulation of histone acetylation of the PD-L1 gene leading to improved and persistent gene expression. The effectiveness of combining HDAC inhibitor with PD-1 blockade for the treatment of melanoma was studied in a murine B16F10 model and compared to control and single-agent therapies, they had a slower tumor development and a higher survival rates (62).

Jhanelle et al. Phase 1/1b study of pembrolizumab plus vorinostat in advanced/metastatic NSCLC concluded that Pembrolizumab plus vorinostat was well tolerated and demonstrated preliminary anti-tumor activity despite progression on prior ICI treatment. Of 30 patients (6 ICI-naive and 24 ICI-pretreated) were evaluable for response, 4 (13%) had a partial response, 16 (53%) had SD, and 10 (33%) had PD for a DCR of 67% without any dose-limiting toxicities (63).

#### Targeted Therapy in Combination With ICIs

##### CDK4/6 Inhibitors

CDK inhibitors are a class of naturally occurring molecules that belong to the Cyclin-dependent kinase inhibitors family INK4 and specifically inhibit the CDK4/6 proteins. By dephosphorylating the retinoblastoma tumor suppressor protein, CDK4/6 inhibitors block the G1 to S phase transition of the cell cycle, resulting in cell cycle arrest in tumor cells. Teo ZL et al. showed that a combination of CDK4/6 and PD1 blockade resulted in intensifying tumor growth inhibition. Increased antigen presentation by tumor cells, stimulation of effector T lymphocyte activation, and decreased proliferation of immunosuppressive Treg cells are among the various mechanisms suggested (64, 65). The immune checkpoint pathway is majorly implicated with the increased immune response upon inhibition of CDK4/6 inhibition. The results of a phase Ib clinical trial combining abemaciclib and pembrolizumab in ER-positive HER2-negative women at a 16-week interim study, MBC demonstrated that this combination is secure, with an ORR of 14.3% (66).

In the JPCE trial, 25 hormone receptor-positive Her2 negative metastatic breast cancer patients were treated with a combination of Abemaciclib and Pembrolizumab. In this trial, the partial response (PR) rate at 16 weeks was 14.3% compared to 6.8% seen in the MONARCH-1 trial which used single-agent abemaciclib. There were no complete responses in either trial. In both experiments, the incidence of stable disease was 60.7% in JPCE and 60.6% in MONARCH-1. In the JPCE experiment, fewer patients developed a progressive disease (17.9% and 25.0%). At the early assessment, the disease control rate (DCR) in JPCE was 75%, while in MONARCH-1 it was 67.4%. As of 17-04-2020, the study is still ongoing and results are yet to publish (66).

##### Anti-Angiogenic Drugs

**Figure 2:**
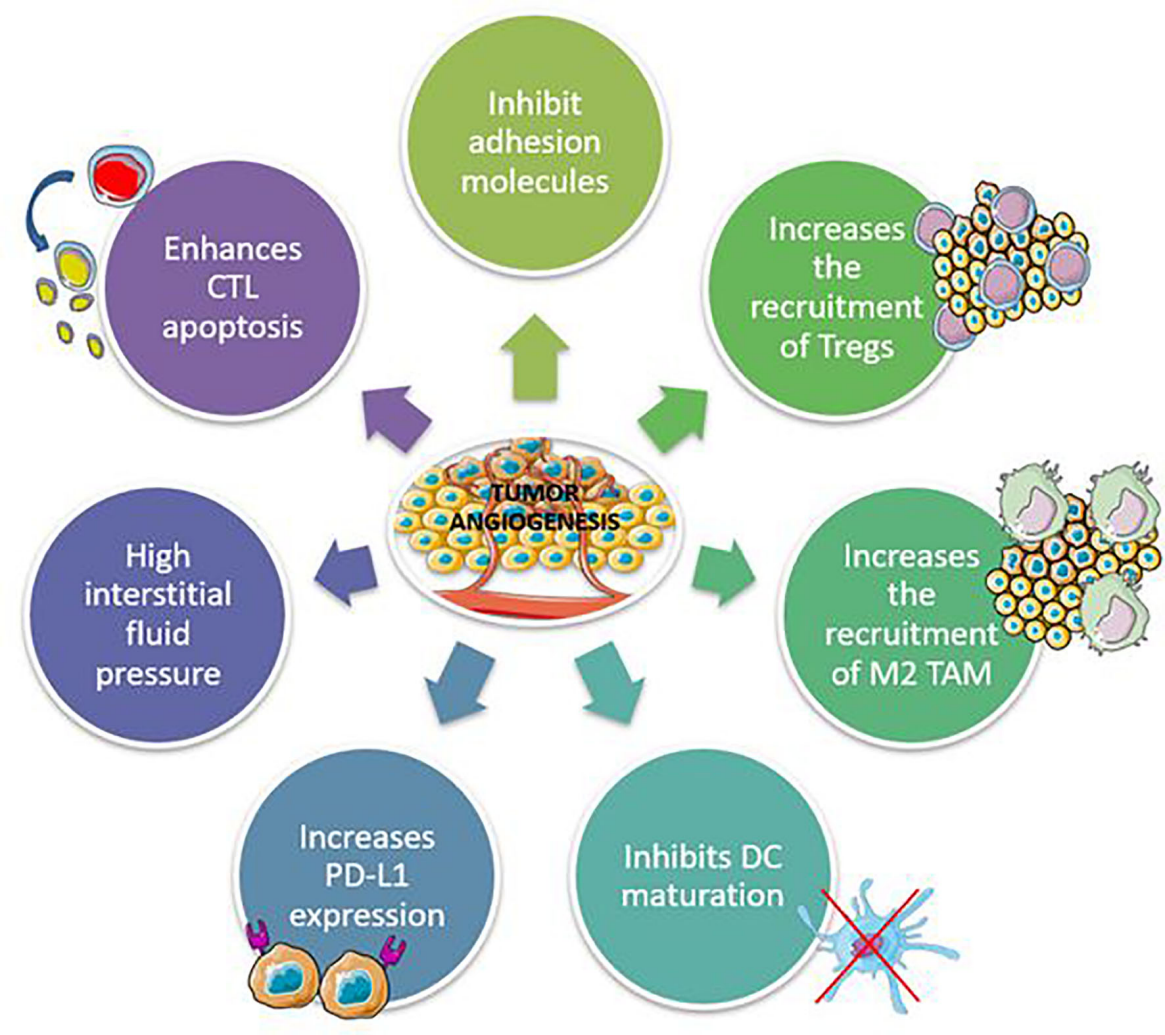
Mechanisms involved in the development of immunosuppressive tumor microenvironment by tumor angiogenesis. CTL, Cytotoxic T lymphocytes; TAM, Tumor-associated macrophages.

Anti-angiogenic drugs work by inhibiting platelet-derived growth factor receptors (PDGFRs) and fibroblast growth factor receptors, which target vascular endothelial growth factor (VEGF)/VEGF2 or other small molecules involved in angiogenic and proliferative pathways (FGFRs). Preclinical studies have shown that abnormal tumor vasculature promotes immunosuppressive TME, which can be reversed with anti-angiogenic therapies (67, 68). Inflammatory mediators such as cytokines and immune cells promote and control angiogenesis, which in turn may affect the microenvironment (**Figure 2**) (69). Anti-angiogenic agents can thus activate the immune system, and immunotherapy can also have an anti-angiogenic effect, therefore they can act synergistically on the tumor.

Results of the phase 3 IMpower150 (NCT02366143) study reported in 2018 showed significant improvement in OS of patients treated with a combination of Atezolizumab, Bevacizumab along with chemotherapy in treatment-naïve metastatic non-squamous non–small-cell lung cancer patients. A total of 800 patients were given either atezolizumab plus bevacizumab plus carboplatin plus paclitaxel therapy (ABCP group) or bevacizumab plus carboplatin plus paclitaxel therapy (BCCP group) (BCP group). The study found that the ABCP community had slightly longer progression-free survival (PFS) and overall survival (OS) (median PFS of ABCP vs. BCP: 8.3 vs. 6.8 months; hazard ratio: 0.61, 95% CI: 0.52 to 0.72) (median OS of ABCP vs. BCP: 19.2 vs. 14.7 months; hazard ratio: 0.78, 95% CI: 0.52 to 0.72). CI ranges from 0.64 to 0.96) (70).

Keynote-426 (NCT02853331), an open-label, phase 3 trial, which randomly assigned 861 patients with previously untreated advanced clear-cell renal-cell carcinoma to receive pembrolizumab (200 mg) intravenously once every 3 weeks plus axitinib (5 mg) orally twice daily (432 patients) and single-agent sunitinib (50 mg) orally once daily for the first 4 weeks of each 6-week cycle (429 patients) which disclose that after a median follow-up of 12.8 months, the estimated %age of patients who were alive at 12 months was 89.9% in the pembrolizumab–axitinib group and 78.3% in the sunitinib group (hazard ratio for death, 0.53; 95% confidence interval [CI], 0.38 to 0.74; P<0.0001) and median progression-free survival was 15.1 months in the pembrolizumab–axitinib group and 11.1 months in the sunitinib group (hazard ratio for disease progression or death, 0.69; 95% CI, 0.57 to 0.84; P<0.001) (71).

##### Inhibitors of DNA Damage Repair

Synthetic lethal impact on cancer cells with a dearth in homologous recombination (HR) is induced by Poly ADP-ribose polymerase (PARP) inhibition. In addition, PARP inhibitors (PARPi) can enhance Th1-skewing immunity and modulate the immune microenvironment, as well as promote the priming of anti-cancer immune responses (72). As the immunological effect of PARPi is multifaceted, it might favor an increased efficacy of ICI treatment and boost the cancer-immunity cycle. Olaparib is a first-in-class PARP inhibitor for the treatment of adult patients with germline BRCA mutated advanced ovarian cancer, breast cancer, fallopian tube cancer, and peritoneal cancer. As discussed the immunological effects of PARP inhibitors are multifaceted (73), which includes

1. PARPi-mediated catastrophic DNA damage; elevated neoantigens and tumor immunogenicity are generated by the accumulation of chromosome rearrangements
2. PARPi-mediated DNA damage could enhance the recruitment and infiltration of T cells into the tumor *via* activating the cGAS-STING pathway
3. PARPi-induced double-strand breaks could directly upregulate PD-L1 by the ATM-ATRChk1 pathway which is independent of the IFN pathway

All these mechanisms reprogram the immune microenvironment to an immune-supportive environment. The maximal benefit of these above-mentioned immunomodulatory mechanisms of PARPi can be achieved in combination with ICIs. Various studies are ongoing, including preclinical as well as clinical trials using the combination of PARP inhibitor + anti-VEGF + ICIs and a combination of PARP inhibitor + checkpoint kinase1 (CHK) + ICIs in cancer (74, 75).

In metastatic ovarian cancer, a recent ESMO update of the MEDIOLA phase I/II open-label, multicenter analysis evaluating the combination of Olaparib and durvalumab in patients with advanced solid tumors who harbor BRCA mutations revealed a median PFS of 15.4 months. Median OS data was not mature yet. 83.5% of patients were still alive after a median follow-up of 23.7 months (76).

##### Other Small Molecule Inhibitors

Several multiple tyrosine kinase inhibitors have shown enhanced action with ICIs. Sorafenib, regorafenib, and Lenvatinib are some of the drugs which have demonstrated superior effect with ICIs in hepatocellular carcinoma. Hepatocellular carcinomas generally arise from an immunosuppressive environment (77). Since it must interact with a variety of foreign antigens, the liver suppresses immune responses to toxins and antigens draining from the enteric circulation. Because of the progressive nature of the disease and the tolerogenic characteristics of the liver, the HCC TME has immunosuppressive characteristics (77–79). HCC exploits this immune tolerance to initiate and promote HCC carcinogenesis and progression. Even though the TME is highly immunosuppressive there are reports which show that ICIs have benefits in the treatment of HCC (78–80). Most of these successful attempts of ICI usage in HCC were demonstrated in combination with multiple tyrosine kinase inhibitors such as sorafenib and regorafenib or when pretreated with these small molecule inhibitors (78–80). However, the exact mechanisms of their additive activity are yet to be proven.

Other small molecule inhibitors include drugs which target PI3K and MAPK pathway. Both the PI3K-Akt-mTOR and the RAS/RAF/MEK/MAPK pathways have been implicated in the control of PD-L1 expression in previous studies (81, 82). Vemurafenib, a BRAF inhibitor, can enhance T cell antigen expression in melanoma and thus stimulate T cell immune responses. By the expression of anti-apoptotic proteins, the PI3K-Akt-mTOR pathway is also involved in resistance to T cell-mediated killing. A more recent study reported better tumor immune infiltration and more anti-tumor immune control in a CD8 T-cell-dependent way when the dual blockade of BRAF and MEK combined with PD-1 inhibitors (83). The experimental evidence presented above may be used to justify clinical trials of BRAF and/or MEK inhibition in conjunction with ICIs. Many studies are ongoing to test these hypotheses and some of the study results are promising. In a study, the selective targeting of PI3K–γ with a small molecule inhibitor IPI-549 (NCT02637531) showed that it could restore the TME and overcome resistance to ICIs by inducing cytotoxic T cell-mediated tumor regression (84). This discovery paves the way for the appealing technique of combining PI3Kis and ICIs to prevent immune checkpoint blockade resistance. One or two agents targeting the PI3KAkt- mTOR pathway could be a possible combination partner for immune checkpoint blockade (85), but the most effective combination is not defined.

Cabozantinib a multikinase inhibitor whose targets include MET, AXL, and VEGFR2 in combination with Nivolumab showed better response rate, delayed disease progression, and extended survival in the CheckMate -9ER trial (86). CheckMate -9ER (NCT03141177) is an open-label, randomized, multi-national Phase 3 trial that investigates Cabozantinib+Nivolumab combination in treatment naïve advanced or metastatic renal cell carcinoma. However, 3 drug combinations of Cabozantinib+Nivolumab+Ipilimumab for the same indication were discontinued.

Phase 1/2 (Checkmate-040) study of doublet therapy (Cabozantinib+Nivolumab) in advanced HCC reported 19% ORR, 75% DCR and median PFS 5.4 months. DOR was 8.3 months with a median OS of 21.5 months in the doublet. Interestingly, the triplet therapy (Cabozantinib+Nivolumab+Ipilimumab) showed comparatively better ORR (29%), DCR (83%), median PFS (6.8 months), and both DOR and median survival were not reached in triplet therapy. However, triplet therapy is associated with an increased risk of treatment-related toxicity (Grade 3 or 4) compared to doublet (71% vs 47%) (87).

##### Bruton’s Tyrosine Kinase Inhibitors

Bruton’s tyrosine kinase (BTK), bone marrow-expressed kinase (BMX), redundant resting lymphocyte kinase (RLK), and IL-2 inducible T-Cell kinase are all members of the Tec kinase family (ITK). BTK is primarily expressed on B cells, though not exclusively, and ITK is primarily expressed on T cells (88). Ibrutinib is a small molecule that not only targets BTK, which is required for malignant B cell survival, but also blocks ITK, which shifts the Th1/Th2 balance and improves antitumor immune activity. The combination of ibrutinib anti-tumor activity and ICIs showed synergism in preclinical models and its positive therapeutic outcome does not result from the direct action against tumor cells, but rather from their activity on the immune system (89). Ibrutinib activates CD8+ T cells, lowers MDSC cytokine secretion, and modifies the inhibitory activity of immune checkpoint molecules like the PD1/PD-L1 and CTLA-4 axis on tumor-infiltrating lymphocytes (89, 90). Many clinical trials are testing this hypothesis and most of them are being tried in lymphoma patients. However, there is limited data in solid tumors and one study showed no benefits of adding ibrutinib along with ICIs (durvalumab) in pretreated solid tumors such as pancreatic cancer (ORR- 2%, Median OS- 4.2 months), breast cancer(ORR- 3%, Median OS- 4.2 months) and NSCLC (ORR- 0%, Median OS- 7.9 months) (91).

##### Selective Estrogen Down Regulators

Fulvestrant is a selective ER down regulator (SERDs), a class of ERα antagonists. Fulvestrant treatment aids in overcoming various resistance mechanisms by ERα down-regulation. Several preclinical studies have proposed various mechanisms by which SERDs exert an anti-tumor response. It alters the TME by increasing Th1 immune response, decreasing Tregs, and inhibiting estrogen-mediated cell proliferation pathways (92, 93). Results of recent translational research indicate that SERDs with strong anti-estrogen activity such as Fulvestrant and potentially other anti-estrogens (94) can augment the action of ICIs to inhibit breast cancer progression. As a result, combining anti-estrogens with ICI may be an effective treatment technique for both ER-positive and potentially ER-negative or treatment-resistant breast cancers, greatly increasing the use and life-extending effects of these drugs to improve patient survival (95).

#### Combination of Multiple Checkpoint Inhibitors

Recently, many studies are focusing on the combination of immunotherapy agents which target multiple pathways in cancer. It is proposed that targeting multiple checkpoints can increase the activity of each other and thereby overcoming each monotherapy’s limitations. The combination of CTLA-4 and PD 1/PD-L1 blockade has exhibited antitumor efficacy in preclinical models (96). The logic of combining multiple checkpoint inhibitors is that they have different mechanisms of action, with anti – CTLA-4 mainly acting in the lymph node compartment which is responsible for restoring the induction and proliferation of activated T cells, and with anti –PD-1 mainly acting at the periphery of tumor site, preventing the neutralization of cytotoxic T cells by PD-L1 expressing tumor and plasmacytoid dendritic cells in the TME (97).

In the RCT checkmate 067 trial in advanced melanoma, patients with PD-L1-positive tumors had the same median progression-free survival (14 months) when they were given nivolumab alone or in combination. However, in patients with PD-L1–negative tumors, ipilimumab plus nivolumab had a longer progression-free survival (11.2 months) than nivolumab alone (5.3 months). The major disadvantage of combination immunotherapy is the increase in grade 3 or grade 4 toxicities. The updated data in 2017 reported that only patients with no expression of PD-L1 benefited from the combination and the recent update in 2019 added that the %age of patients experiencing a complete response continued to increase, with complete response rates at five years of 22% for combination therapy and the proportion of patients alive and treatment-free was 74% in nivolumab/ipilimumab combination (98).

Based on ORR and DOR in phase I/II CheckMate-040 trials, FDA has approved Nivolumab-Ipilimumab combination in Hepatocellular carcinoma in March-2020. In the study, 16 of 49 patients (33%) treated with nivolumab in combination with ipilimumab responded to treatment after a minimum of 28 months of follow-up (95% CI, 20-48). Four patients (8%) had a full response (CR) and 12 people (24%) had a partial response (PR). DORs ranged from 4.6-30.5 months, with 88% lasting at least 6 months, 56% lasting at least 12 months, and 31% lasting at least 24 months (99, 100).

Updated result of Phase 3 Checkmate-214 trial (Ipilimumab+Nicolumab) in treatment naïve renal cell carcinoma after 42-month median follow-up showed very promising result with CR in 10% patients, OS rate and ORR as 52% and 42% respectively and DOR was not reached (101).

FDA granted fast track designation for investigation of the combination of Balstilimab PD-1 inhibitor and Zalifrelimab a CTLA-4 inhibitor for the treatment of patients with relapsed or refractory metastatic cervical cancer. The FDA designates an investigational drug for fast track review in order to speed up the production of drugs that treat severe or life-threatening conditions and address an unmet medical need. Combination of Balstilimab/Zalifrelimab exhibit 26.5% objective response rates (ORR) vs 11.4% in Balstilimab monotherapy (102).

#### Microbiome

Microorganisms live on our skin, in our lungs, and in our intestines, accounting for a large portion of our cellular, metabolic, and genetic mass (103). The microbiota is the collective term for the species that make up this population, while the microbiome is the collective term for their genetic material. Inflammatory bowel disease, autism, asthma, and obesity have all been linked to the intimate relationship between microbiota composition, metabolic function, and immune system development and regulation (104). When a combination of broad-spectrum antibiotics (i.e., ampicillin plus colistin plus streptomycin) and single-agent imipenem elicited antitumor effects of CTLA-4 monoclonal antibody (mAb) as a result of microbiota impairment, the relationship between microbiota and ICIs was demonstrated. Antibiotic use between two months before and two months after the start of immunotherapy has been linked to a worse prognosis in patients treated with anti–PD-1/PD-L1 mAb (105). It is suggested that microbiota can influence dendritic cell (DC) maturation and activation. Several studies have reported the influence of bacteria such as *Akkermansia mucinphila*, *Bacteriodetes*, and *Firmicutes* in augmenting the response of ICIs. Creating a favorable microbiome using fecal transplantation in patients on ICIs is also being widely studied.

**Figure 3:**
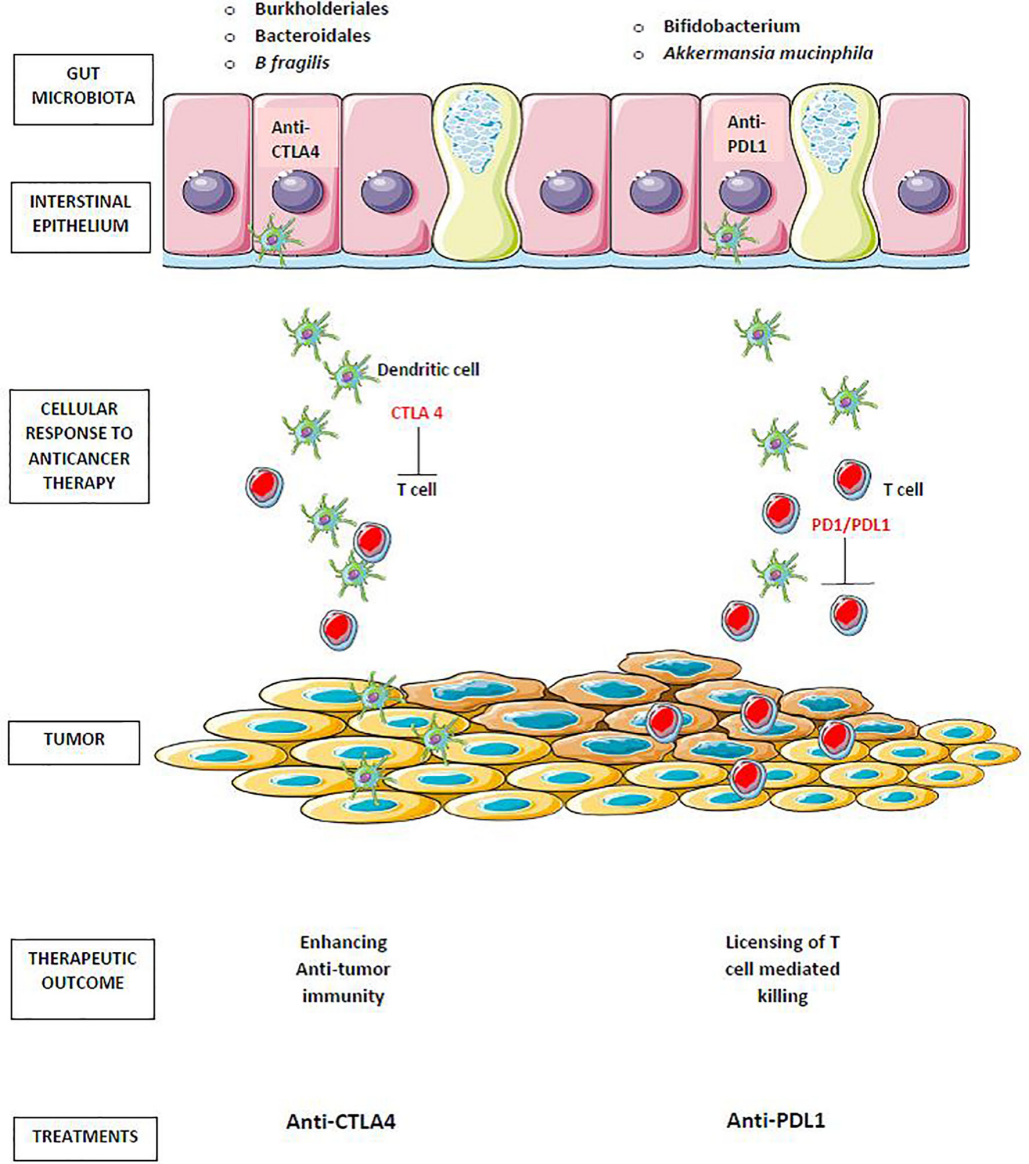
Gut microbiota in cancer immunotherapy. Translocation of *Burkholderiales* and *Bacteroidales*, enhances antitumor immunity of anti–cytotoxic T-lymphocyte antigen 4 (anti-CTLA-4). The antitumor effect of anti–PD-1/PD-L1 therapy enhanced by the preexisting antitumor immunity that is particularly effective in mice harboring intestinal *Bifidobacterium* spp and *Akkermansia mucinphila*.

Anti–cytotoxic T-lymphocyte antigen 4 (anti-CTLA-4) immunotherapy causes mucosal damage and the translocation of Burkholderiales and Bacteroidales bacteria, which promote anti-commensal immunity as an adjuvant to anti-tumor immunity and are necessary for a positive response to therapy. Anti–PD-1/PD-L1 therapy, which does not harm the gut epithelia, requires pre–existing antitumor immunity, which is particularly effective in mice with intestinal Bifidobacterium spp (106) as portrayed in **Figure 3**.

#### Oncolytic Virus

Oncolytic viruses also appear to be an additional therapeutic agent for the management of cancer. These agents specifically target and kill cancer cells leaving the normal cells undamaged. Oncolytic viral infection triggers anti-cancer immune responses, which boost the effectiveness of checkpoint inhibition (107). Oncolytic viruses elicit anti-tumor immunity by promoting DC maturation and function. Improved DC maturation and function is because of the ability of oncolytic viruses in transferring genes encoding IFN-α, GM-CSF, and other cytokines. Breakdown of tumor cells induced by the oncolytic virus results in the release of DAMPS which further facilitates anti-tumor immune response. DAMPS include cell surface proteins, membrane proteins, and nucleic acid (108). FDA has approved an intralesional oncolytic immune therapy known as *Talimogene Laherparepvec* (T-VEC) for stage3b and stage 4 melanoma. A phase II trial comparing a combination of ipilimumab and T-VEC with ipilimumab monotherapy showed better ORR in the combination arm (39% vs. 18%; p < 0.02). Phase1b study of T-Vec combination with Pembrolizumab in melanoma patients reported no dose-limiting toxicity with an ORR of 62% and a CRR (complete response rate) of 33% (109). Many studies including a phase III trial is underway (110). Increased PD-L1 expression and distant inflammation from the injection sites are reported after T-VEC from the analysis performed before the administration of anti-PD1 antibodies. Similarly, several other oncolytic viruses are being evaluated under clinical studies in combination with ICIs as well as a single agent. HF 10 virus included in the HSV family, Tasadenoturev (DNX-2401), members of the Poxviridae family (Pexa-Vec), Coxsackieviruses (CVA 21) are some of the oncolytic viruses which have shown promising results in combination with ICIs in melanoma, GBM, liver cancer, etc. (111–119).

#### Radiotherapy

It is known that radiation induces DNA damage-induced apoptosis and recent evidence suggests that it stimulates tumor antigen release and exerts an immune-mediated tumor response (120). Radiotherapy has been stated to enhance immunosuppressive TME *via* (1) Treg proliferation, M2 polarization of TAMs, and MDSC accumulation by increasing HIF 1 alpha transcription (2) activating latent TGF beta in the tumor, which converts CD4+ T-cells to Tregs and polarizes TAMs into M2 phenotypes (121, 122). Some studies demonstrate the abscopal effect after radiation therapy distinctly from the treatment effects of immunotherapy alone (123). The abscopal effect is described as a phenomenon in the management of metastatic cancer by which localized radiation activates systemic antitumor effects and eradicates distant metastasis. There are several Phase 1 and Phase 2 clinical trials are ongoing in patients with metastatic NSCLC, metastatic head, and neck cancer, and metastatic castration-resistant prostate cancer, etc. (124–126).

#### Other Drugs

Many other drugs are found to have an additive or synergistic effect with ICIs such as Inducible T cell Costimulatory (ICOS) Receptor Agonist, Nectin 4 directed therapies, and anti-IL-6 vaccine. Enfortumab vedotin, a Nectin-4-directed antibody and microtubule inhibitor conjugate, was recently granted accelerated approval by the FDA for the treatment of adult patients with locally advanced or metastatic urothelial cancer who have previously obtained a programmed death receptor-1 (PD-1) or programmed death-ligand 1 (PD-L1) inhibitor and platinum-containing chemotherapy in the neoadjuvant/adjuvant, locally advanced or metastatic setting. A recent study shows that the combination of Enfortumab Vedotin and pembrolizumab demonstrates encouraging efficacy with a tolerable and manageable safety profile in an early phase trial for locally advanced or metastatic urothelial carcinoma. A phase 1b study reported by C J Hoimes et al. states that of the 29 enrolled patients, preliminary confirmed ORR per RECIST 1.1 was 62% by investigators, including a 14% CR rate. The DCR was 90%. (NCT03288545) (127).

GSK609 is an anti-Inducible T cell Co-Stimulator (ICOS) receptor agonist antibody that is used to treat cancers with various histologies. The first in man Phase 1 studies are ongoing as a single agent and along with Pembrolizumab. In patients with previously treated, PD-1/L1 naive HNSCC, preliminary evidence suggests that the combination of GSK609 and pembrolizumab has promising antitumor activity and a manageable safety profile (NCT02723955) (128).

Promising results are reported with anti-Her2 antibody (trastuzumab) in combination with ICIs in patients with HER2-positive metastatic esophagogastric cancer. A study reported a median PFS of 13·0 months (95% CI 8·6 to not reached), and a median OS of 27·3 months with a median DOR of 9.4 months (129). Phase 3 Keynote-811 trial is ongoing and expected to get a detailed result in combining pembrolizumab and trastuzumab (130). Similarly, clinical trials are ongoing to evaluate the response of other anti-tumor antibodies such as rituximab (anti-CD20) in combination with pembrolizumab in recurrent follicular lymphoma and DLBCL (NCT02446457) and cetuximab (anti EGFR)+ ICI combination in recurrent/metastatic HNSCC (NCT03494322) and RAS wild type metastatic colorectal cancer (NCT04561336).

A very recent study published by Chan LC et al. found that JAK1, activated by IL-6 phosphorylates Tyr112 of programmed death-ligand 1 (PD-L1), causing STT3A, an endoplasmic reticulum-associated N-glycosyltransferase, to catalyze PD-L1 glycosylation and maintain PD-L1 stability. In animal models, targeting IL-6 with an IL-6 antibody resulted in synergistic T cell killing when paired with anti–T cell immunoglobulin mucin-3 (anti–Tim-3) therapy. In hepatocellular carcinoma patient tumor tissues, there was a positive association between IL-6 and PD-L1 expression. These findings point to the use of anti–IL-6 and anti–Tim-3 as a marker-guided therapeutic strategy (131).

#### Other Drugs in Combination With ICIs

There are a few drugs that are not used in cancer therapy however have been widely studied in combination with ICIs. The ultimate aim of using these drugs as adjuncts to ICIs is to create an immune stimulatory TME. Most of these studies are still in the preclinical setup but some of them are in clinical studies and show favorable outcomes (**Table 3**).

**Table 3:** Few clinical trials listed in Clinical trials.gov which uses non-cancer drugs combination strategies with ICIs as of 17-04-2020.

Adjunct Therapy	ICI used	Study Title	Clinical Trial Reference	Phase	Status
Rosiglitazone Metformin	Nivolumab or Pembrolizumab	A Phase II Clinical Trial of Anti-PD-1 mAb Therapy Alone or With Metabolic Modulators to Reverse Tumor Hypoxia and Immune Dysfunction in Solid Tumor Malignancies	NCT04114136	Phase 2	Not yet recruiting
Tadalafil	Nivolumab	Window of Opportunity Trial of Nivolumab and Tadalafil in Patients With Squamous Cell Carcinoma of the Head and Neck	NCT03238365	Phase 1	Active
Tadalafil	Nivolumab	A Phase II Study of Tadalafil and Pembrolizumab in Recurrent or Metastatic Head and Neck Squamous Cell Carcinoma	NCT03993353	Phase 2	Not yet recruiting
Paricalcitol	Pembrolizumab	A SU2C Catalyst Randomized Phase II Trial of the PD1 Inhibitor Pembrolizumab With or Without a Vitamin D Receptor Agonist Paricalcitol in Patients With Stage IV Pancreatic Cancer Who Have Been Placed in Best Possible Response	NCT03331562	Phase 2	Active
Galunisertib	Nivolumab	A Phase 1b/2 Dose Escalation and Cohort Expansion Study of the Safety, Tolerability and Efficacy of a Novel Transforming Growth Factor-beta Receptor I Kinase Inhibitor (Galunisertib) Administered in Combination With Anti-PD-1 (Nivolumab) in Advanced Refractory Solid Tumors (Phase 1b) and in Recurrent or Refractory NSCLC or Hepatocellular Carcinoma	NCT02423343	Phase 2	Active
Losartan, FOLFIRINOX, SBRT	Nivolumab	A Randomized Phase 2 Study of Losartan and Nivolumab in Combination With FOLFIRINOX and SBRT in Localized Pancreatic Cancer	NCT03563248	Phase 2	Recruiting
Epacadostat	Nivolumab	A Phase 1/2, Open-Label, Dose-Escalation, Safety, Tolerability, and Efficacy Study of Epacadostat and Nivolumab in Combination With Immune Therapies in Subjects With Advanced or Metastatic Malignancies (ECHO-208)	NCT03347123	Phase 2	Active
Propranolol	Pembrolizumab	A Phase Ib/II Study of Propranolol With Fixed-Dose Pembrolizumab in Patients With Unresectable Stage III and Stage IV Melanoma	NCT03384836	Phase 2	Recruiting
Aspirin	Atezolizumab	A Phase II Study of the Anti-PDL1 Antibody Atezolizumab, Bevacizumab and Acetylsalicylic Acid to Investigate Safety and Efficacy of This Combination in Recurrent Platinum-resistant Ovarian, Fallopian Tube or Primary Peritoneal Adenocarcinoma	NCT02659384	Phase 2	Recruiting
Aspirin or Celecoxib	Pembrolizumab	PD-1 Antibody Combined With COX Inhibitor in MSI-H/dMMR or High TMB Colorectal Cancer: a Single-Arm Phase II Study	NCT03638297	Phase 2	Recruiting
Celecoxib	Nivolumab	NICE-COMBO: An Open-Label Phase II Study Combining Nivolumab and Celecoxib in Patients With Advanced “ Cold “ Solid Tumors	NCT03864575	Phase 2	Not yet recruiting
Celecoxib	Nivolumab Ipilimumab	Nivolumab, Ipilimumab, and COX2-inhibition in Early Stage Colon Cancer: an Unbiased Approach for Signals of Sensitivity (NICHE)	NCT02431208	Phase 1	Active

##### Drugs Targeting Myeloid-Derived Suppressor Cells

The key targets in the TME are considered to be MDSCs. Research demonstrates that diminished immunotherapy efficacy is associated with the presence of MDSCs in the TME (132, 133). MDSCs play a vital role in the immunosuppression of TME. These cells possess a strong immunosuppressive potential by representing a heterogeneous population of immature myeloid cells. Antitumor reactivity of NK cells and T cells have also been inhibited by MDSCs. They also encourage angiogenesis, the formation of pre-metastatic niches, and the recruitment of other immunosuppressive cells including regulatory T cells (134). Many commonly prescribed drugs such as rosiglitazone, cimetidine, tadalafil, etc. have shown preclinical benefits in targeting MDSCs (135). Even though the mechanism behind these drugs is different, the goal is to create an immune stimulatory TME inhibiting MDSCs. Examples of these classes of drugs with their proposed mechanism of action include:

- Rosiglitazone: Reduction of early MDSC accumulation
- PDE5 antagonist (Tadalafil and sildenafil): Reduction of MDSC levels, reduction of arginase and iNOS production
- Vitamin D: Stimulate differentiation of immature myeloid cells into DCs
- Amiloride: Inhibit MDSC suppressive capacity *via* reduced exosome secretion in preclinical studies
- Cimetidine: Reduction of MDSC expansion by induction of apoptosis and inhibition of NOS and ARG-I expression in preclinical studies

Paricalcitol a synthetic vitamin D analog has been used along with ICIs in some of the pancreatic clinical trials and showed a favorable response compared to conventional therapies. In a phase 2 trial using paricalcitol in combination with nivolumab, gemcitabine, nab-paclitaxel, and cisplatin in patients with untreated metastatic pancreatic ductal adenocarcinoma (PDAC), of the 25 patients enrolled thus far, OS has been recorded as 15·3 months, with an underlying ORR of 83% (NCT02754726).

A J Lugibuhl et al. conducted a study using Tadalafil Nivolumab combination in preoperative Head and Neck squamous cell cancer (HNSCC) showed 50% of patients having a pathologic treatment effect (TE)  > 21% in 4 weeks. In the context of PD-1 blockade, tadalafil enhanced T lymphocyte and myeloid cell infiltration. 45 patients were treated with the combination regimen, and a preliminary assessment of pathologic treatment effect was performed on post-treatment specimens, revealing that 25% of patients had no treatment effect (TE), 25% had 1-20% TE, 41% had 21-99% TE, and 9% had CR (NCT03238365) (136).

##### Drugs Targeting Tregs

Regulatory T cells (Tregs) play a pivotal role in maintaining immune homeostasis and self-tolerance. They also play a negative role in inducing efficacious anti-tumor response. There is substantial evidence that depleting Tregs or inhibiting Treg function improves antitumor effects (137, 138). The suppressive activity of Treg cells is exhibited by various mechanisms including secretion of inhibitory cytokines such as TGF beta, IL10, and IL35; inhibition of APC maturation through the CTLA-4 pathway; and expression of granzyme and perforin which kills effector T-cells (139, 140). TGFβ inhibitors and IDO inhibitors are currently explored along with checkpoint inhibitors in various cancer models such as melanoma, NSCLC, head, and neck, renal and bladder cancer, etc.

###### TGFβ Inhibitors

Transforming growth factor (TGF) is a cytokine that induces Tregs and mediates immunosuppression in the TME. Therefore the strategy of blocking the recruitment of Tregs by TGFβ inhibitors has shown benefits in some of the preclinical trials (141). T-cell sequestration away from the tumor mass is caused by the expression of a fibroblast TGF response signature (F-TBRS) in peritumoral fibroblasts. Immune checkpoint blockade with PD-1/PD-L1 inhibitors is ineffective in the absence of physical proximity between cytotoxic T cells and tumor cells. Pharmacological inhibition of TGFβ reverses such immune exclusion and promotes CD8+ T cell penetration into tumors. TGF inhibition combined with immune checkpoint blockade maximizes tumor regression, with several tumor-bearing mice showing complete remission (142–145). Active TGF-signaling in the peritumoral stroma, especially in patients with low tumor-infiltrating T cells in the tumor parenchyma, resulted in a lack of response to atezolizumab (anti-PD-L1 mAb) in metastatic urothelial cancer, according to a study (146). Galunisertib a TGFβ inhibitor is in various clinical trials in combination with ICIs in NSCLC, pancreatic cancer, urothelial cancer, etc. Besides, galunisertib is also in a clinical trial for advanced refractory solid tumors along with ICIs (NCT02423343).

###### IDO Inhibitors

In response to interferon-gamma, tumor cells and MDSCs express IDO. Recent research has found that IDO activity is important for FoxP3 Treg activity (147) and MDSCs, leading to suppression of the activity of T cells and NK cells (148). Several IDO inhibitors are being investigated in combination with CTLA-4 and PD-1 inhibition based on promising preclinical results. Epacadostat is an example of an IDO inhibitor that is being studied along with ICIs in clinical trials. COX-2 inhibitors which are another class of drugs have been found to have potential use as an adjunct with ICIs to treat immunosuppressive tumors that constitutively express indoleamine 2,3-dioxygenase (IDO1) (149–151). In the absence of IDO activity, recent studies have discovered that Tryptophan 2, 3-dioxygenase (TDO), another essential enzyme in the kynurenine pathway, plays a compensatory role. As a result, a dual inhibitor of IDO and TDO is currently being investigated in preclinical studies to achieve maximum inhibition of the kynurenine pathway and alleviate tumor immune suppression (152).

##### Drugs Targeting the Renin-Angiotensin System (RAS)

**Figure 4:**
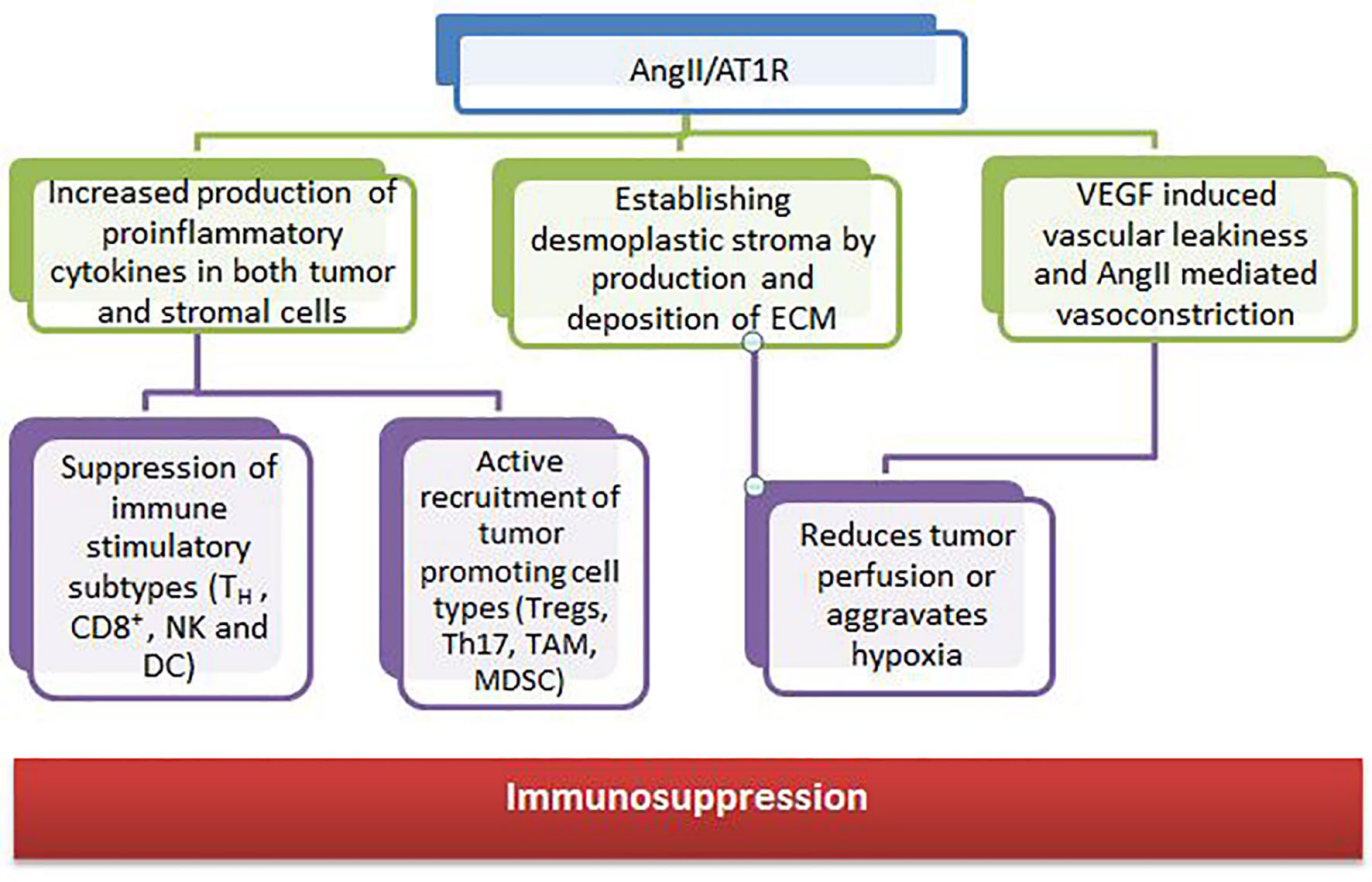
Contribution of angiotensin II and angiotensin receptor to the tumor microenvironment.

RAS components are also expressed in many cell types of the TME, such as endothelial cells, fibroblasts, monocytes, macrophages, neutrophils, dendritic cells, and T cells (153, 154). Growth and dissemination can be facilitated or hindered by RAS signaling in these cells which have been shown to affect cell proliferation, migration, invasion, metastasis, apoptosis, immunomodulation, angiogenesis, cancer-associated inflammation, and tumor fibrosis/desmoplasia (155, 156). The angiotensin II and angiotensin receptor (AngII/AT1R) axis regulates the tumor stroma and contributes to an immunosuppressive microenvironment through various mechanisms (**Figure 4**).

Several clinical studies have also shown that RASi may have favorable effects in a wide range of malignancies. The gain in survival is tumor type– and stage-dependent and ranged from 3 months (advanced NSCLC) to more than 25 months (metastatic RCC) in retrospective studies (157). Drugs such as losartan are already in clinical trials as an adjunct with ICIs in many cancers.

##### Drugs Targeting the Autonomic Nervous System (ANS)

Lately, the role of the nervous system, in particular, the sympathetic nervous system has been observed in immune response regulation. Normally, the response of acute stress is beneficial whereas chronic stress is detrimental due to the suppression of effector immune cell activity while enhancing the immunosuppressive cell activity (158). Beta-blockers have been shown to have significant benefits when used along with ICIs. Data have shown that decreasing adrenergic stress by housing mice at thermoneutrality or treating mice housed with β-blockers at cooler temperatures reverses immunosuppression and remarkably increases responses to ICIs (159). Various pre-clinical, as well as clinical studies, have shown improved efficacy when ICIs are used along with a β blocker (159–161).

##### Metformin

Metformin has shown antitumor properties in various studies (162, 163). Metformin prevents gluconeogenesis in the liver by regulating the adenosine monophosphate-activated protein kinase (AMPK)/liver kinase B1 (LKB1) pathway. The AMPK/LKB1 pathway controls the cell cycle by regulating protein synthesis and cell proliferation by adjusting the amount of energy needed by the cells (164). Cancerous cells are inhibited and apoptosis is induced as a result of this regulation of the cell cycle and proliferation. Metformin was found to target CD8+ tumor-infiltrating leukocytes in a sample (TILs). Metformin also prevents the production of immune tolerance to cancer cells by inhibiting the synthesis of unfolded proteins, activating the immune response to cancer cells, inhibiting the expression of CD39/73 on MDSCs, and inhibiting the expression of CD39/73 on MDSCs. Metformin prevents exhaustion of CD8+ TILs, thus increasing production of various cytokines such as TNF-α, TNF-γ, IL-2 and reversing immunosuppression. Some studies in NSCLC have reported that metformin exhibits cytotoxic effects as well (165–167). Preclinical and clinical studies are ongoing to explore the efficacy of metformin as an adjunct to ICIs.

An interim safety analysis of Durvalumab Metformin combination in Head and Neck squamous cell carcinoma proved safe in part 1 of the study which contains only 6 patients and enrollment for part 2 with more patients is currently ongoing (NCT03618654) (168).

### Future Implications

**Figure 5:**
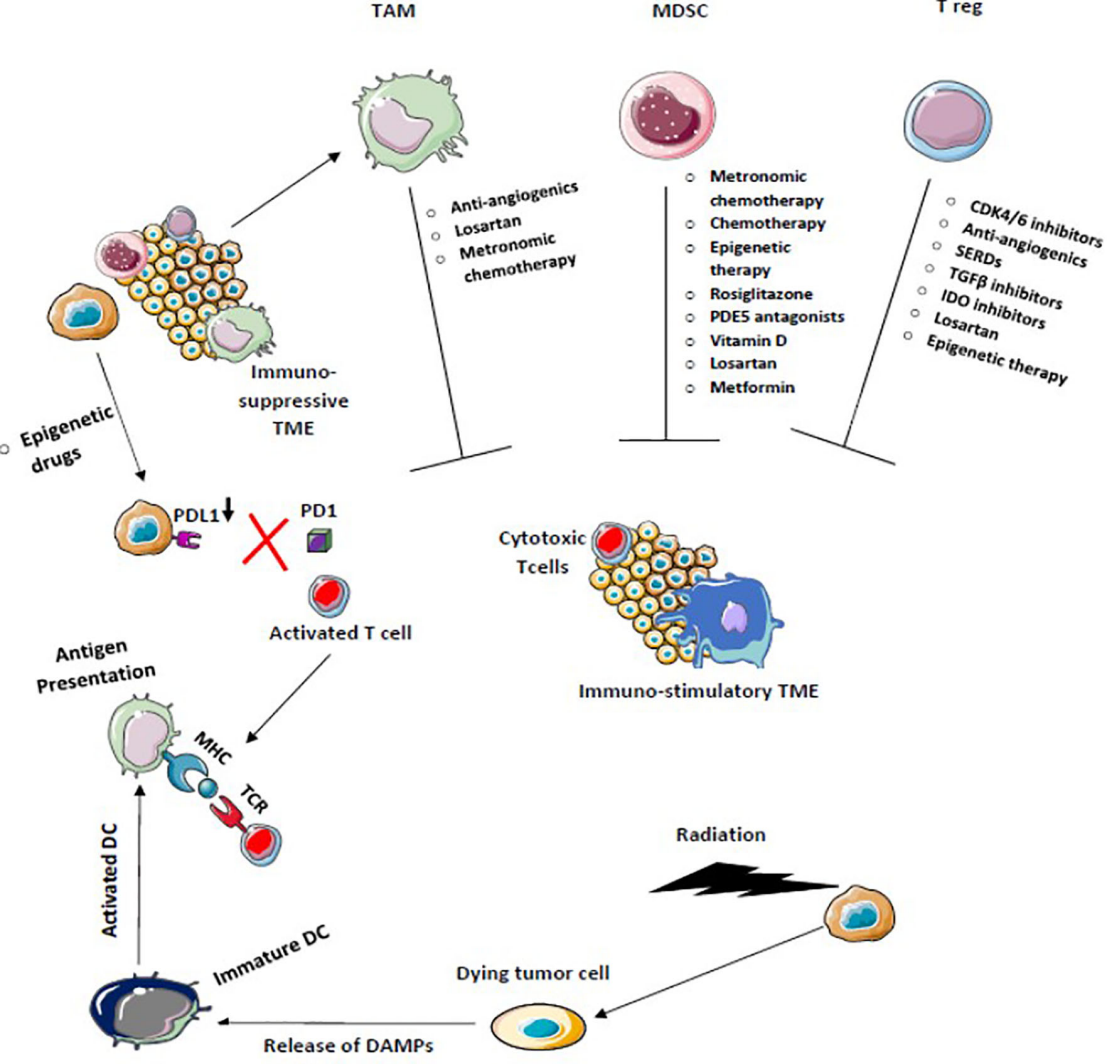
Chemotherapy, Radiotherapy, and Epigenetic therapy which facilitates immunogenic cell death increase antigen presentation enhances cross-priming of dendritic cells, and decreases PDL-1 expression on tumor cells activates the antitumor immune response. Drugs that target Tregs, tumor-associated macrophages (TAM), and myeloid-derived suppressor cells block immunosuppression and enhance T effector T cell function to convert immunosuppressive tumor microenvironment into the immune-stimulatory environment.

**Figure 6:**
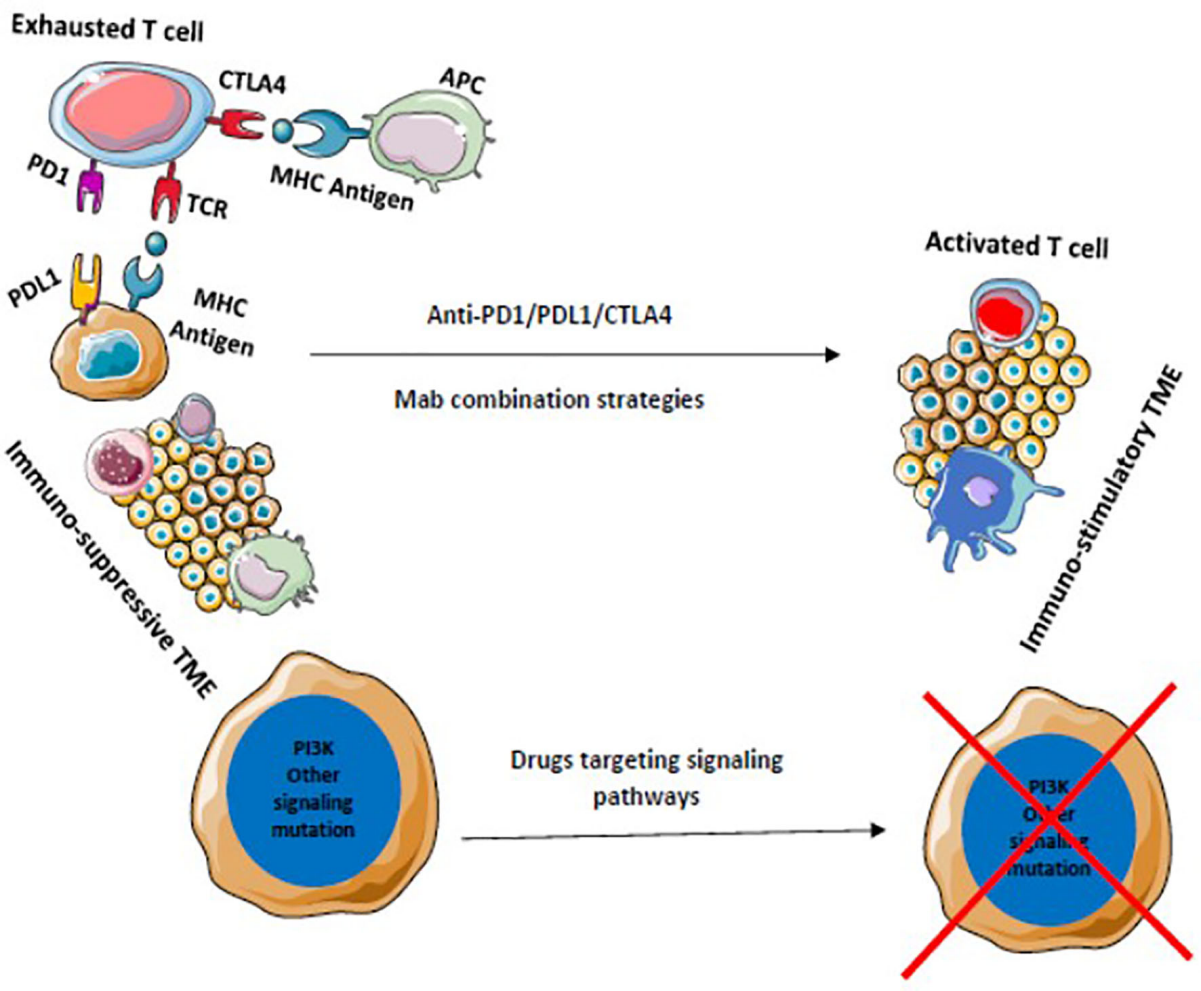
Drugs that target multiple signaling pathways kill tumor cells and stimulates immune cells to provide a durable adaptive immune response. Blocking of multiple immune checkpoints stimulates immune responses against tumors.

Various other modalities are being explored to achieve maximum benefit from ICI therapy. Newer checkpoint targets are being analyzed such as LAG-3, TIM-3, TIGIT, VISTA, or B7/H3. Moreover, stimulatory checkpoint pathway agonists such as GITR, 4-1BB, OX40, ICOS, CD40, or molecules targeting TME components like TLR or IDO are under investigation (169, 170). Inhibiting multiple pathways along with ICIs is another ongoing topic of research. Combination of multiple checkpoint inhibitors and triplet or doublet therapy with adjunct drugs are being clinically validated. Improving TME to produce an immune stimulatory environment is being widely explored and drugs targeting these immune suppressive cells such as MDSC, TAM, etc. are being developed (**Figures 5** and **6**). Targeting mast cells is another approach that is currently being studied. Some of the recent studies have reported that intra-tumoral mast cell levels rose as the tumor progressed, and this independently predicted a shorter OS (171). It was found that CXCL12-CXCR4 chemotaxis results in the accumulation of these tumor-infiltrating mast cells. Intra-tumoral mast cells induced by cancer strongly express PD-L1 in both dose-dependent and time-dependent manner (172). This mechanism can be explored in the future to develop adjunct drugs with ICIs.

### Translational Perspectives

As the spectrum of combinatorial immunotherapy strategies widens, the demand for robust preclinical models is at an all-time high. Combination strategies with ICIs require stalwart preclinical models for precise repurposing of the partner drugs. Murine models generally used previously may not be helpful in immuno-oncology because of fundamental differences in the immune composition between mice and humans. The introduction of a patient-derived humanized xenograft model (Hu-PDX) is a very promising strategy in immuno-oncology. Hu-PDXs are mouse cancer models where tissue or cells from a patient’s tumor are implanted into a humanized mouse which contains an immune system identical to humans (173). This knowledge of the Hu-PDX model further propelled research in immuno-oncology and enlightened the concept of co-clinical trial. In a co-clinical trial model, the clinical trial is coupled with a preclinical trial which provides valuable information to the corresponding clinical trial (174). Simultaneous treatment of the Hu-PDX model of a patient enrolled in a clinical trial with new agents helps in further understanding of underlying mechanisms, evaluating novel combination strategies, and identification of potential biomarkers. These advantages of co-clinical trials will ensure the swift transition of preclinical data to the clinic which will be of great value to precision medicine and immuno-oncology.

Besides Hu-PDX models, patient-derived tumor three-dimensional organoid models are also an exciting proposition for co-clinical trials (175). Organoids (and cancer-derived organoids) are three-dimensional tissue-like cellular clusters made from tissue or tumor-specific stem cells that imitate *in vivo* (tumor) characteristics such as cell heterogeneity (176). The major limitation is the inability to create a tumor-associated immune system in these models. Despite these limitations, organoid cultures accurately mimic the *in vivo* response of patient-derived xenograft models, as demonstrated in recent studies using bladder cancer-derived organoids by Pauli et al. and Lee et al. (177, 178).

## Discussion

Our review shows that the majority of the adjuncts, which vary from chemotherapy, targeted therapy, radiation, immune-modulators, or even other forms of immunotherapy when combined with ICIs could provide far superior results than previously achieved. Adjunct drugs/modalities with favorable and unfavorable responses are distinctly illustrated in our review.

Maike Trommer, Sin Yuin Yeo et al. showed abscopal effects of RT in up to 29% of the patients when combined with anti PD1 immune check point inhibition (123). Huaqin Yuan et al. showed that Axitinib which is a known antiangiogenic agent could have an immune effect through its inhibition of MDSCs, STAT 3 pathway, and stimulation of CD8 T cells. This effect of Axitinib could enable us to use it as an adjunct along with checkpoint inhibition (179). A R Folgueras et al. showed that epigenetic modification could enhance immune surveillance in epithelial tumors. They found that HDAC inhibition could enhance the immunogenicity of epithelial tumors (180).

Similarly, David Roulois, Helen Loo Yau et al. found that hypomethylating agents may mount an antitumor response by inducing viral mimicry by endogenous transcripts (181). Recent studies have even used a triple-drug combination to augment antitumor immunity. Alexandra S. Zimmer, Erin Nichols et al. found a combination of PD1 inhibitor, Parp inhibitor, and antiangiogenic agent to be well tolerated and effective in a phase 1 study (75). Joseph A. Califano, Zubair Khan et al. found that even non-cancer drugs like tadalafil can have an immune-stimulating effect. Their work showed that tadalafil can reverse the resistance to immune recognition in head and neck cancer when patients were administered daily tadalafil (182).

Even though ICI’s have shown tremendous promise especially in tumors refractory to standard treatments, their response rates are rarely beyond 15-20% (183). Translational research is of utmost value in this scenario and may help to further harness and improve the potential of immunotherapy.

Firstly, standard immune biomarkers like PDL1 levels, MSI status may not hold good when adjuncts are being used along with ICIs. Hence the need to develop newer biomarkers such as tumor mutational burden, tumor-infiltrating lymphocytes, miRNA studies, establishing ‘foreignness’ of neoantigens, HLA variations, N/L ratio, etc. so that a larger %age of patients can reap the benefits of ICIs. Artificial intelligence, neural networks, virtual clinical trials, and patient-derived xenograft models may be required to determine which patients may benefit from ICIs, to learn about the variation in the durability of response among patients, and identify those who may benefit from re-challenge of ICIs.

Secondly, the dose of adjuncts to be administered with ICIs would need to be determined. Ideally, an immune stimulatory’ dose is required which may or may not be the same as the standard dose used otherwise. Once proof of concept is established, the ICI-adjunct combination could be used in tumor agnostic/basket trials thus further tapping into unknown potentials of ICIs. Thirdly, although the mechanisms of ICI-adjunct combinations have been broadly defined, the exact additive/synergistic mechanisms need to be determined through well-constructed trials and translational research before they can become part of standard treatment. Fourthly, adverse effects of ICI-adjunct combination could be a lot greater than when either of the agents is used singly. Moreover, failure of combination strategies in the first-line may result in a lack of availability of drugs for second-line use. Hence proper analysis of adverse events and determining risk: benefit ratio would be required before such combinations become part of routine practice.

Finally, the emerging role of the microbiome and its effect on ICI combinations needs to be addressed and evaluated as it may play a major role in immune-modulation and effectiveness of such combinations. The above-addressed points all form a major portion of T0 and T1 phases of translational research which could greatly widen our understanding and applicability of ICIs and their combinations.

## Conclusion

The efficacy of ICIs is highly variable and only a few patients are benefited from them. To address this issue there are many ongoing translational studies to target the resistant mechanisms of ICIs. The main principle is to enhance the TME to an immune stimulatory type from an immune-suppressive one. In this review, we have evaluated available literature which included drugs used as adjuncts in clinical trials along with ICIs. Many drugs have shown significant benefits and some have shown a mixture of both favorable and unfavorable outcomes. There is conflicting evidence for most of the above-mentioned adjuncts such as metformin, Vit D, etc. From the available adjuncts, the selection of the best adjunct for the patients on ICI treatment is still a dilemma. Not only the selection of adjuncts but also the dose for the desired property is also questionable. It might require a dose that is different than used normally and needs to be further evaluated. Therefore, the need of conducting co-clinical trials or translational studies in this field is immense. We believe that recent advances in preclinical models such as humanized xenograft models, invitro modeling using 3D bioprinting, etc. may give further insight into this problem.

## Author Contributions

HV: Data collection, scientific writing, proof reading, and concept. VS: Scientific writing, proof reading, and concept. BT: Data collection and scientific writing. SM: Concept, proof reading, and scientific writing. RN: Proof reading and project overseer. All authors contributed to the article and approved the submitted version.

## Conflict of Interest

All authors are affiliated with the company HealthCare Global Enterprises Ltd.
